# Acute Ileocolitis

**Published:** 1906-07

**Authors:** Albert Conrey

**Affiliations:** Baltimore, Md.


### ABSTRACTS.

### Acute Ileocolitis.

By ALBERT CONREY, M. D., Baltimore, Md.

(*Author's Abstract.*)

THE onset of this case was sudden with some vomiting, but with much pain and fever and thin green stools partly fecal and containing undigested food. In a few hours the discharges were composed entirely of blood and mucus, preceded by pain and tenesmus. The stools were about thirty minutes apart, each one containing less than a teaspoonful. Prolapsus ani was present and there was exquisite tenderness all along the colon; temperature 104°, and prostration was very marked.

*Treatment.*—As the pathological process seemed to be principally in the lower half of the colon, it was decided to treat the case altogether by injections. Glycothymoline, one ounce to the pint, was the only drug used and was administered in the following manner: the solutions were made hot and at least a gallon administered at one time. It was injected high into the colon through a long rectal tube and early in the disease repeated twice daily. Before using the glycothymoline the colon was always cleansed with about two gallons of hot sterile water. After each irrigation four ounces of a somewhat stronger solution of glycothymoline was introduced high into the bowel and its escape prevented by compression of the buttocks. In a few days blood disappeared from the stools which at the same time became less frequent and pain and tenesmus ceased. Mucus persisted in the stools for not quite two weeks.

